# Friendship Conflict, Drinking to Cope, and Alcohol-Related Problems: A Longitudinal Actor-Partner Interdependence Model

**DOI:** 10.1177/21676968211060945

**Published:** 2022-03-11

**Authors:** Sean P. Mackinnon, Michelle E. Tougas, Ivy-Lee L. Kehayes, Sherry H. Stewart

**Affiliations:** 1 3688 Dalhousie University, Halifax, NS, Canada

**Keywords:** alcohol use/abuse, coping, friendship, transitions to adulthood, peers

## Abstract

Drinking to cope with negative affect is a strong predictor of alcohol-related problems. We hypothesized that the association between friendship conflict and alcohol-related problems would be mediated by coping-with-depression motives in emerging adults’ close friendships. We used a 4-wave, 4-month longitudinal self-report survey design measuring friendship conflict, coping motives, and alcohol-related problems from 174 same-sex friendship dyads. Participants were recruited from Nova Scotia, Canada between September 2016 and February 2019. Participants had a mean age of 18.66 (*SD* = 1.17) and were 66.1% female. Data were analyzed using multilevel structural equation modeling. Coping-with-depression motives mediated the link between conflict and alcohol-related problems at the between- and within-subject levels. Unexpectedly, coping-with-anxiety motives was an additional mediator at the within-subjects level. Interventions for emerging adults’ problem drinking should consider the influence of friendship conflict and its impact on emerging adults’ tendencies to drink to cope with both depression and anxiety. Materials/Syntax: https://osf.io/krs3v/

Emerging adults (∼18–29 years old; Arnett et al., 2014) have the highest prevalence of alcohol use, with 82.8% of Canadian emerging adults drinking in the past year (Canadian Centre on Substance Use and Addiction, 2017). Among Canadian post-secondary students, 35% report consuming at least five or more drinks in one sitting in the last 2 weeks, and 49.5% report at least one alcohol-related problem over the past 12 months (American College Health Association, 2019). The most common alcohol-related problems experienced by post-secondary students are having a bad time (33.0%), noticing a change in personality (31.7%), neglecting responsibilities (27.6%), missing a day (or part-day) of school or work (25.6%), and being unable to do homework or study for a test (22.1%) (Neal et al., 2006). The present study tests whether friendship conflict and drinking to cope predict alcohol-related problems in friendship dyads using a 4-wave, 4-month longitudinal design.

## Drinking Motives Theory

Young people drink alcohol for a variety of motives, some of which are riskier than others. The motivational model of alcohol use (Cooper, 1994; see also Cooper et al., 2016) describes two underlying dimensions of the consequences that young people seek from drinking alcohol: (1) positive vs. negative reinforcement; and (2) internal versus external motivational sources. More specifically, the desired outcomes of drinking may involve pursuing a positive outcome (e.g., pleasurable arousal) or avoiding a negative outcome (e.g., avoiding depression), and achieving sought after internal (e.g., mood manipulation) or external (social approval) consequences. Combining these underlying drinking motivation dimensions results in four individual drinking motives: social (positive reinforcement, external), enhancement (positive reinforcement, internal), conformity (negative reinforcement, external), and coping (negative reinforcement, internal) (Cooper, 1994; Cooper et al., 2016).

Each of the four drinking motives are associated with specific alcohol-related outcomes (see Cooper et al., 2016). Drinking to cope (i.e., drinking to alleviate negative affect) is unique in that it is directly related to alcohol-related problems (e.g., problems with school; Cooper et al., 1995) even after accounting for alcohol consumption levels. Further research exploring the four-factor model of alcohol-use motives found that subdividing coping motives into two distinct factors to create a five-factor model was a better fit for assessing drinking motivations in emerging adults (Grant, Stewart, O'Connor, et al., 2007). To create the five-factor model, coping motives were divided into coping with anxiety motives (CAM) and coping with depression motives (CDM), which both uniquely predicted distinct alcohol-related outcomes. Coping with anxiety motives showed cross sectional and CDM longitudinal associations with alcohol-related problems (Grant, Stewart, O'Connor, et al., 2007). Further exploration is needed of factors, such as friendship conflict, that may lead to each of these coping motives and in turn to alcohol-related problems.

## Friendship Conflict and Alcohol

Conflict within friendships may be an important trigger of both negative emotions and of drinking in young people given that friendships are central to the lives of emerging adults (McNamara Barry et al., 2015). In a large cross-sectional study of 1074 emerging adult friend dyads, Boman et al. (2013) found that friendship dyads where both friends engage in binge drinking and cannabis use were characterized by increased conflict. One possible explanation for this link is that friendship conflict may trigger heavy drinking in both friendship dyad members. Studies of young friendship dyads have examined whether friendship conflict leads to negative emotions. Schwartz–Mette et al. (2021) found that positive friendship quality longitudinally predicted lower depression in cross-lagged panel models for adolescent friend dyads; however, friendship conflict was generally unrelated to depressive symptoms in this sample. In contrast, Chow et al. (2015) found that friendship discord was associated with increased depressive symptoms in university student friendship dyads. One possible reason for the discrepant results is the difference in developmental stage (adolescence vs. emerging adulthood). The more transitory nature of friendships during emerging adulthood (Laursen & Bukowski, 1997) and the tendency in this developmental stage to shift focus from friendships to romantic relationships (McNamara Barry et al., 2009) might give rise to increased friendship conflict in the transition from adolescence to emerging adulthood (Camirand & Poulin, 2019) and to result in negative emotions that could trigger coping drinking. Thus, we focused our investigation on emerging adulthood. Another difference between Chow et al. (2015) and Schwartz–Mette et al. (2021) pertains to the operationalization of conflict—the item content of Chow et al. (2015) focused much more on criticism, dominance, and exclusion than the measure used by Schwartz–Mette et al. (2021). The present study utilized measures that tap friendship conflict in a manner consistent with Chow et al. (2015).

In the present study, we conceptualized conflict as a dyadic variable—for example, mutually expressed anger, hostility, and/or communicative disengagement that happens within a given relationship. This contrasts with research on social negativity, which conceptualizes anger, hostility, and criticism as an intra-individual variable (Ibarra–Rovillard & Kuiper, 2011). Thus, our operationalization of conflict measures it as an inherently interpersonal process (i.e., relational conflict between people, rather than a measurement of intra-individual hostility), as we believe it is dyadic conflict between friends, not the act of being hostile toward a friend per se, that predicts drinking to cope.

## Replication Target

Using a 4-wave, 4-week longitudinal design with 100 romantic dyads, Lambe et al. (2015) found that the relationship between conflict and alcohol-related problems was mediated by coping motives. Specifically, only CDM (and not CAM) motives mediated the conflict-to-alcohol problems association at the within-subjects level when both motives were entered as mediators in a single model (Lambe et al., 2015). These results were found for actor effects (how an individual influences their own behavior), but not for partner effects (how a partner influences an individual’s behavior). Therefore, increases in conflict within a romantic relationship predicted increases in CDM drinking motives which, in turn, predicted increases in alcohol-related problems in the same individual.

Lambe et al. (2015) has important implications for the treatment of drinking problems. Specifically, it implies that romantic relationship conflict might be a distal predictor of alcohol problems—thus, addressing relationship conflict could have downstream improvements for alcohol-related problems. Nonetheless, romantic relationships represent only one facet of an emerging adult’s social network and potential relationship conflicts. Thus, we wanted to see if the findings of Lambe et al. (2015) generalized to another important type of relationship in the lives of emerging adults, namely friendships. We sought to conceptually replicate and extend Lambe et al. (2015) with three primary methodological changes. The first change was to recruit same-sex friends instead of romantic couples. At the emerging adult developmental stage, young people increasingly turn to peers for social interactions and social support, with these friendships influencing many areas of their lives (Buote et al., 2007; Lewis et al., 2015). Ruptures in these friendships may have significant consequences on feelings of anxiety and depression (Chow et al., 2015, which may trigger coping-motivated drinking and in turn alcohol-related problems. Thus, the current study investigated the mediational role of each of the coping motives (CDM and CAM) in explaining the relationship between friendship conflict and alcohol-related problems in both emerging adult actors and their friends.

The second methodological change was to change from a 4-wave, 4-week design to a 4-wave, 4-month design. A primary difficulty in longitudinal research is to find the right time lag for the causal processes under study. A weakness of the Lambe et al. (2015) study was that the 1-week measurement occasions were short, and did not necessarily allow sufficient time for conflict, motives, and alcohol problems to change over time. Moreover, short time lags can result in restricted variance for comparatively rare events, such as conflict and alcohol problems. Thus, we increased the lag between measurement occasions to increase the variation in our studied constructs. The third change was a move from in-person data collection to online data collection. This change was for efficiency and pragmatics. Otherwise, the methods of the present study were virtually identical to Lambe et al. (2015).

## Objectives

Using a longitudinal, 4-month, 4-wave design, this study examined the association between friendship conflict and alcohol-related problems. This association was further explored with CDM and CAM motives as potential mediators of the association between friendship conflict and alcohol-related problems. Moreover, we investigated actor and partner effects. Partner effects may be expressed directly, where a friend’s drinking motive may impact an individual’s own alcohol-related problems. Partner effects may also be expressed indirectly, wherein a friend’s drinking motive predicts an individual’s own alcohol-related problems, which in turn may influence the individual’s own alcohol-related problems. Given the longitudinal nature of the study, data were analyzed using multilevel structural equation modeling (SEM) (Preacher et al., 2010). Structural equation modeling explores between-subject variance, the portion of variance that stays consistent across the 4 months, as well as within-subject variance, the portion of variance that changes from month to month.

**Figure 1.**
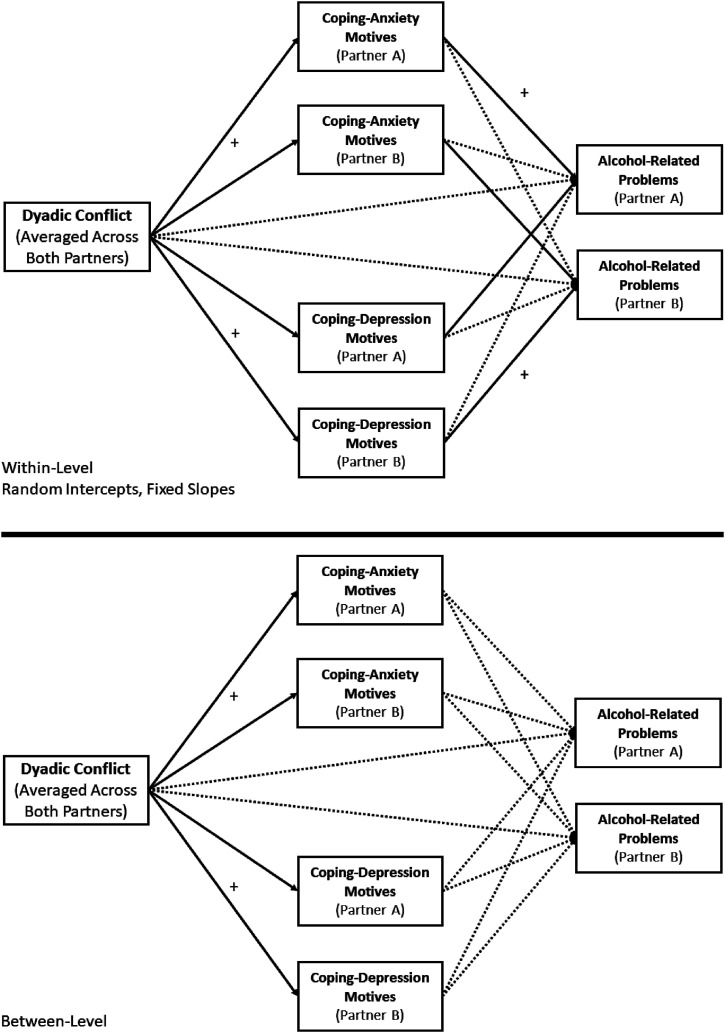
Figure depicting confirmatory hypotheses (solid lines) and exploratory hypotheses (dotted lines). *Note.* Rectangles indicate manifest variables. Single-headed arrows indicate paths. Residual covariances are omitted on this conceptual diagram to reduce clutter. Black lines indicate hypothesized paths based on Lambe et al. (2015) and the expected direction (+, or positive). Dotted lines represent exploratory research questions for other paths included in the model.

Given that this research aimed to replicate and extend the work of Lambe et al. (2015), the hypotheses and research questions were formulated from their findings on the relationship between conflict, coping motives, and alcohol-related problems in romantic couples. Our hypotheses were also informed by prior findings linking friendship conflict to depression (Chow et al., 2015) and conflict to alcohol outcomes (Boman et al., 2013) in emerging adult friendship dyads. In our confirmatory model replicating Lambe et al. (2015), we tested the same model, and derived our predictions from the findings of that study. A visual depiction of the model and our predictions for the direction of relationships derived from Lambe et al. (2015) can be found in Figure 1. These predictions can be summarized as follows:

1) Friendship conflict would have an indirect effect on alcohol-related problems through CDM motives, meaning that friendship conflict would lead to drinking to cope with depression, which in turn would lead to alcohol-related problems. Specifically, we expected these findings to hold for actor effects at the within-subjects level.
2) Friendship conflict would lead to drinking to cope with anxiety at both the between- and within-subject levels.

Other paths in Lambe et al. (2015) were inconclusive. Thus, for other paths in Figure 1, we did not have a priori predictions, and these tests were considered exploratory.

1) Would friendship conflict have an indirect effect on alcohol-related problems through CDM motives for partner effects and/or at the between-subjects level?
2) Would friendship conflict have an indirect effect on alcohol-related problems through CAM motives for actor and/or partner effects at either the between- and/or within-subject levels?

## Method

### Participants

Participants were required to be same-sex friends who consume alcohol together and had known each other for a year or less. Participants were included in the study if they consumed 12 or more alcoholic drinks in the past year, were between the ages of 18–25 years old, and at least one of the friends was a first-year undergraduate student. These requirements were listed in our recruitment materials. All participants met these criteria when screened via email prior to arrival at the lab. All participants were recruited from Nova Scotia, Canada. These data were previously analyzed in three prior studies, one examining the relationship between drinking motives and alcohol quantity/frequency (Kehayes et al., 2021), another on extraversion and drinking similarity (Nogueira–Arjona et al., 2019), and a third on the validity of informant-reported drinking motives (Kim et al., in press); our study made secondary use of these data.

Participants were 348 undergraduate students from 174 same-sex friendship dyads. Individual participants had an average age of 18.66 years (*SD* = 1.17), were 66.1% female, 79.3% Caucasian, and 84.8% were university students. They were recruited from Dalhousie University and the surrounding community. Participants reported hearing about the study through flyers (37.9%), word-of-mouth (25.0%), class website (20.7%), classroom announcements (17.0%), the Psychology Department participant pool (10.1%), or other sources (15.8%), with some participants reporting more than one source. At Wave 1, the initial point of contact with the lab, the dyads reported an average friendship length of 4.05 months (*SD* = 2.21), with an average face-to-face contact of 19.75 days/month (*SD* = 7.60). At Wave 1, a total of 21.0% friends reported cohabitating together for an average of 2.88 months (*SD* = 1.56). At Wave 1, 85.1% reported experiencing at least one alcohol-related problem in the past month (*M* = 4.14, *SD* = 3.60, range = 0–18).

### Procedure

Data collection for the first wave started in September 2016 and ended February 2019^1^. Data were collected during the Fall and Winter semesters. Participants were recruited using the psychology subject pool, online ads, flyers, and in-class announcements. Interested participants contacted the study administrators via email and were screened for eligibility. Dyads arrived together at the lab and were again screened for eligibility. Participants reviewed the consent form and gave informed consent prior to participation. During the same visit, each participant completed the Wave 1 questionnaire battery online. For three monthly follow-ups, participants were emailed the same questionnaire batteries for Waves 2–4 at 30-day intervals. If participants did not complete a questionnaire the day it was mailed, they were emailed reminders daily for 7 days, with three additional reminders until the end of the 30-day period. Reminders ceased after completion of the questionnaires. The make-up questionnaires evaluated the same 30-day period as the original to ensure that responses would be for the same 30-day period, regardless of when completed. If participants did not complete one of the waves, their data was counted as missing for that wave only. Skip logic was employed, where participants were not asked to complete the drinking motives questionnaire for a given wave if they abstained from alcohol during that month (i.e., an individual cannot have a motive for drinking if they did not drink). Participants were compensated CAD$10.00 or one credit point for each wave that they completed on-time (within a week of the questionnaire being sent). For questionnaires that were completed 8–30 days after originally being sent, participants were compensated with CAD$5.00 or ½ credit point. All participants were debriefed at completion of participation.

### Materials

#### Demographics and friendship

At Wave 1, each participant completed questions about their demographic characteristics (age, sex, and ethnicity) and information about their friendship (friendship length, amount of face-to-face contact, and cohabitation).^2^

#### Conflict

Friendship conflict was analyzed as a composite variable that consisted of the Social Conflict Scale (Abbey & Andrews, 1985), the Partner-Specific Rejection Behaviors Scale (Murray et al., 2003), and the Interpersonal Qualities Scale (Oishi & Sullivan, 2006). Each scale measured friendship conflict in the past month. The Social Conflict Scale consisted of five items (e.g., “Got on your friend’s nerves”) rated on a scale ranging from 1 (*not at all*) to 5 (*a great deal*). The Partner-Specific Rejection Behaviors Scale consisted of seven items (e.g., “I insulted my friend”) rated on a scale ranging from 1 (*strongly disagree*) to 9 (*strongly agree*). The Interpersonal Qualities Scale consisted of five items that rated interpersonal characteristics when in the presence of the study friend (e.g., “moody/irritable”), ranging from 1 (*not at all characteristic*) to 9 (*completely characteristic*). In previous work, each individual scale has exceeded acceptable levels of internal consistency (alphas ranging from 0.75–0.84; Lambe et al., 2015). Averaged subscale totals for each of the three scales were used for descriptive statistics. For analyses, each subscale total was converted to standardized Z scores, which were then summed to create a single conflict composite index score. This composite measure has been shown to possess acceptable psychometric properties with factor analysis showing a single factor with loadings from each of the 17 conflict items ranging from 0.47 to 0.81 (Lambe et al., 2015). This established factorial validity indicated that combining the conflict items into a single factor was appropriate. For the current study, conflict was assessed as a dyadic variable with equal contributions from each member of the friendship (Lambe et al., 2015; Mackinnon et al., 2012).

#### Modified drinking motives questionnaire—Revised

Coping with depression motives and CAM were derived from the 30-day version of the Modified Drinking Motives Questionnaire-Revised (DMQ-R; Grant, Stewart, O'Connor, et al., 2007), a modified five-factor version of the original four-factor drinking motives scale (Cooper, 1994). The DMQ-R is a reliable and valid 28-item measure of five drinking motives: enhancement, social, conformity, CDM, and CAM. As the main focus of this study was to replicate and extend the work of Lambe et al. (2015) with friendship dyads, only CDM and CAM were used in analyses. The CDM scale is composed of nine items (e.g., “to numb my pain”), and the CAM scale is composed of four items (e.g., “to reduce my anxiety”). Participants rated how often they drank for each reason on a scale from 1 (*almost never/never*) to 5 (*almost always*). The Modified DMQ-R has been found to have good to excellent test-retest reliability (intraclass correlation coefficients from 0.61–0.78), adequate to excellent internal consistency (αs from 0.66 to 0.91), and strong factorial validity (Grant, Stewart, O'Connor, et al., 2007).

#### Rutgers alcohol problem index

The 30-day version of the Rutgers Alcohol Problem Index (RAPI) was used to measure alcohol-related problems (White & Labouvie, 1989). The RAPI is a 23-item measure asking about the experience of specific negative consequences (e.g., “caused shame or embarrassment to someone”) due to drinking. Participants rated how often each problem occurred in the specified 30-day timeframe on a scale from 0 (*never*) to 4 (*4 or more times*). The RAPI has been found to have strong test-retest reliability (*r* = .83), and high internal consistency (α = .92) for the total RAPI score (Grant, Stewart, O'Connor, et al., 2007). The individual RAPI items were dichotomized (i.e., 1 = presence, 0 = absence of alcohol-related problem). The dichotomized items were then summed into a single value (possible range 0–23) for analysis (Martens et al., 2007). The dichotomized RAPI shows good psychometric properties including good internal consistency with αs ranging from 0.74 to 0.83 (Martens et al., 2007).

### Self-administered Timeline Follow-Back

Using a self-reported timeline follow-back procedure (Collins et al., 2008), participants reported on their past 30 days of alcohol consumption. Participants reported on the number of alcoholic drinks they consumed each day, with a standard drink defined as 12oz of beer, 5oz of wine, 3oz of fortified wine, or 1.5oz of hard liquor. Total volume of consumption was calculated by summing the total number of drinks consumed over the past 30 days. The Self-administered Timeline Follow-Back (STLFB) provides similar results to more traditional quantity-frequency measures of consumption (Collins et al., 2008).

### Data Analytic Strategy

**Figure 2.**
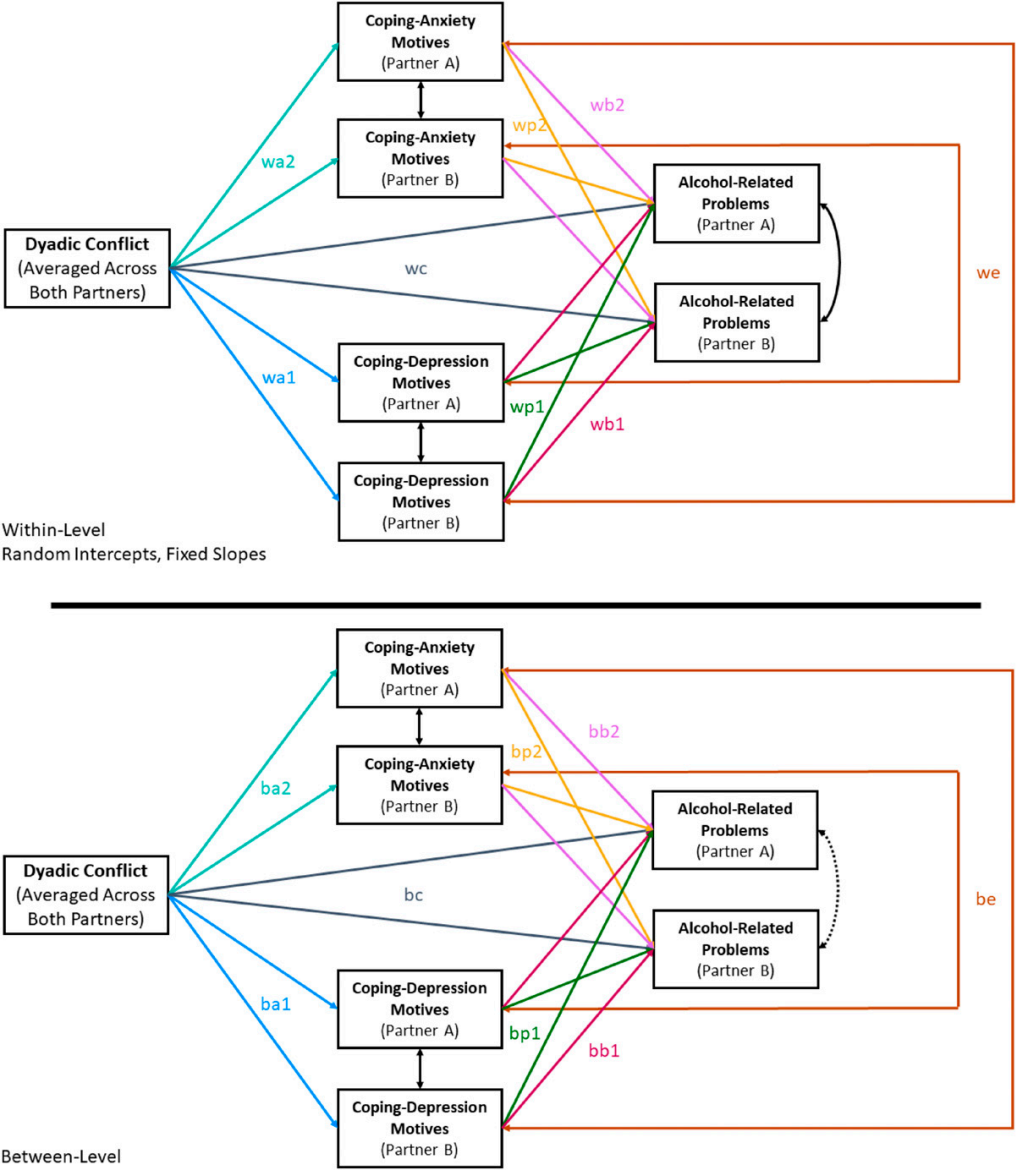
Tested multilevel structural equation model including constraints. *Note.* Pathways that share a color and label (e.g., wa2) were constrained to equality due to indistinguishable dyads. Paths were not constrained to equality across levels (e.g., wa2 and ba2 are not equal to one another). Variance partitioned into between and within levels using latent mean centering. Actor effects are paths wb1, wb2, bb1, and bb2. Partner effects are paths wp1, wp2, bp1, and bp2. Double-headed arrows are correlated residuals to account for non-independence.

Data were analyzed using Mplus 7.0 software using multilevel structural equation modeling (Preacher et al., 2010), combining elements of traditional mediation models and Actor Partner Interdependence models (Kenny & Ledermann, 2010). The tested model is depicted in Figure 2. Data were in wide format for dyads (i.e., separate columns for partner A and B), with dyadic non-independence handled using correlated residuals. Because dyads are indistinguishable, partners were randomly assigned the role of Partner A or B, and paths were constrained to equality as depicted in Figure 2. Data were in long format for the longitudinal component (i.e., one row per timepoint), and non-independence was handled using multilevel modeling. The multilevel model partitioned variance into between- and within-subjects levels using latent mean centering (see Hamaker & Muthén, 2020). We used random intercepts and fixed slopes; this essentially assumes compound symmetry for the longitudinal residual variances (Streja et al., 2017, p. ii80). Missing data was handled using a full information maximum likelihood approach. Significance of indirect effects was calculated using the delta method, as bootstrapping is incompatible with TYPE = TWOLEVEL in Mplus software. Nonnormality of residuals was accounted for by using a robust estimator of fit indices and standard errors (MLR estimator in Mplus). Moreover, because skewness was severe enough that the MLR estimation might still be biased, CDM, CAM, and the RAPI were log_10_ transformed^3^ prior to analysis to deal with positive skew (this also matches analytic procedures used in Lambe et al., 2015).

Our choice to use MLR estimation and log transforms instead of other common alternatives for dealing with violations of the normality assumption was based on pragmatic concerns. Bootstrapping is incompatible with multilevel models in Mplus. Count models would require switching to the WLS estimator which requires listwise deletion for missing data. Moreover, count models make interpretation of mediation models difficult, because indirect effects become conditional on values of X (Geldof et al., 2018). Thus, we did not analyze the data using count models. Finally, using log transforms matches the approach used in the replication target (Lambe et al., 2015).

## Results

Out of four waves of surveys, individuals within dyads completed an average of 3.49 waves (*SD* = 0.93), with 70.7% of individuals completing all four waves. Across waves, 100% of individuals completed Wave 1, 89.9% of individuals completed Wave 2, 85.3% completed Wave 3, and 73.6% completed Wave 4. During the study, three dyads reported ending their friendship at Wave 3, and two dyads at Wave 4. For these dyads, data collected before their friendships ended were included in the analyses, while data collected after the friendships ended were coded as missing (0.01% of wave entries). Data from individual participants who reported abstaining from alcohol for any of the waves were excluded from analyses for only the wave that they abstained from drinking alcohol (5.7% of wave entries). In cases where one partner abstained and the other drank alcohol, we still retained data from the individuals who drank alcohol in analyses. No other data were excluded from analyses. A total of 80.2% of the surveys were completed on time, and 19.8% were completed late (ranging from 1–8 days delayed). Across the four waves, 20.3% of data were missing, with covariance coverage ranging from .76–.91.

Table 1 presents the means and *SD*s of values across all four waves. All values were comparable to the replication target (i.e., within 1 *SD* of the mean), with the exception of the RAPI mean for wave 2, which was still within 2 *SD* of the mean from previous samples (Lambe et al., 2015).^4^ Table 2 presents the within- and between-subject level correlations, intraclass correlations, and reliabilities. At both the between-and within-subject levels, all variables were significantly and positively correlated with one another, with the magnitudes of correlations being greater at the between-subjects level. Intraclass correlations show the percentage of the variance available to be explained at the between-subjects level; the majority of the variance was calculated at the within-subjects level for all variables (i.e., a state-trait with substantial state and trait variance). All measures showed good reliability at both the between-and within-subject levels, with the exception of CAM at the within-subject level. When cluster sizes are small, within-subjects level reliabilities may be underestimated, and reliability cutoff scores are not as well-established in multilevel models (Geldhof et al., 2014). Therefore, despite one low reliability value, we proceeded with the planned analysis. Nonetheless, analyses utilizing CAM have a high degree of measurement error at the within-subjects level, and thus an elevated potential for Type II error.

**Table 1.** Means and Standard Deviations.

Variable	Wave 1		Wave 2		Wave 3		Wave 4		Actual Range Possible Range	
	*M*	*SD*	*M*	*SD*	*M*	*SD*	*M*	*SD*		
RAPI	4.14	3.60	4.04	4.14	3.58	4.08	2.79	3.77	0–23	0–23
CDM	1.42	0.69	1.37	0.59	1.33	0.60	1.38	0.77	1–5	1–5
CAM	1.85	0.83	1.79	0.70	1.73	0.72	1.75	0.84	1–5	1–5
Social conflict	1.63	0.65	1.59	0.65	1.60	0.71	1.50	0.64	1–4.80	1–9
Rejecting behaviors	1.64	0.97	1.66	0.96	1.83	1.16	1.65	1.18	1–9	1–9
Interpersonal qualities	3.08	1.61	2.98	1.60	3.06	1.74	3.07	1.85	1–9	1–9
STLFB	42.89	40.26	31.43	32.34	28.19	29.25	23.98	27.91	0–376	Open-ended numerical

*Note.* Ns vary by variable and wave and range from 197 to 348. *Ms* and *SDs* are shown for the averaged total of the summed dichotomized RAPI items, as well as for the averaged subscale totals for the CDM and CAM motives, and the three conflict variables prior to log_10_ transformation. RAPI = Rutgers Alcohol Problem Index; CDM = coping with depression motives; CAM = coping with anxiety motives. STLFB = Volume of alcohol consumption on the self-reported timeline follow-back measure. Actual range is the minimum and maximum values across all 4 waves.

**Table 2.** Correlation Matrix, Intraclass Correlations, and Reliabilities for Analyses at Between- and Within-Subject Levels.

Variable	1	2	3	4	5
1. Conflict	--	.18***	.15***	.21***	.03
2. CDM	.43***	--	.64***	.38***	.17***
3. CAM	.26*	.79***	--	.35***	.24***
4. RAPI	.33***	.54***	.53***	--	.32***
5. STLFB	.06	.26**	.34	.72	--
ICC	.42	.33	.29	.36	.51
Alpha reliability (within)	.85	.92	.66	.80	--
Alpha reliability (between)	.95	.98	.86	.95	--

*Note.* Between-subject correlations are below the diagonal; within-subject correlations are above the diagonal. CDM, CAM and RAPI were log_10_ transformed. CDM = coping with depression motives; CAM = coping with anxiety motives; RAPI = Rutgers Alcohol Problem Index; STLFB = Volume of alcohol consumption on the self-reported timeline follow-back measure; ICC = intraclass correlation.

*p < .05. **p < .01. ***p < .005.

### Mediation

**Figure 3.**
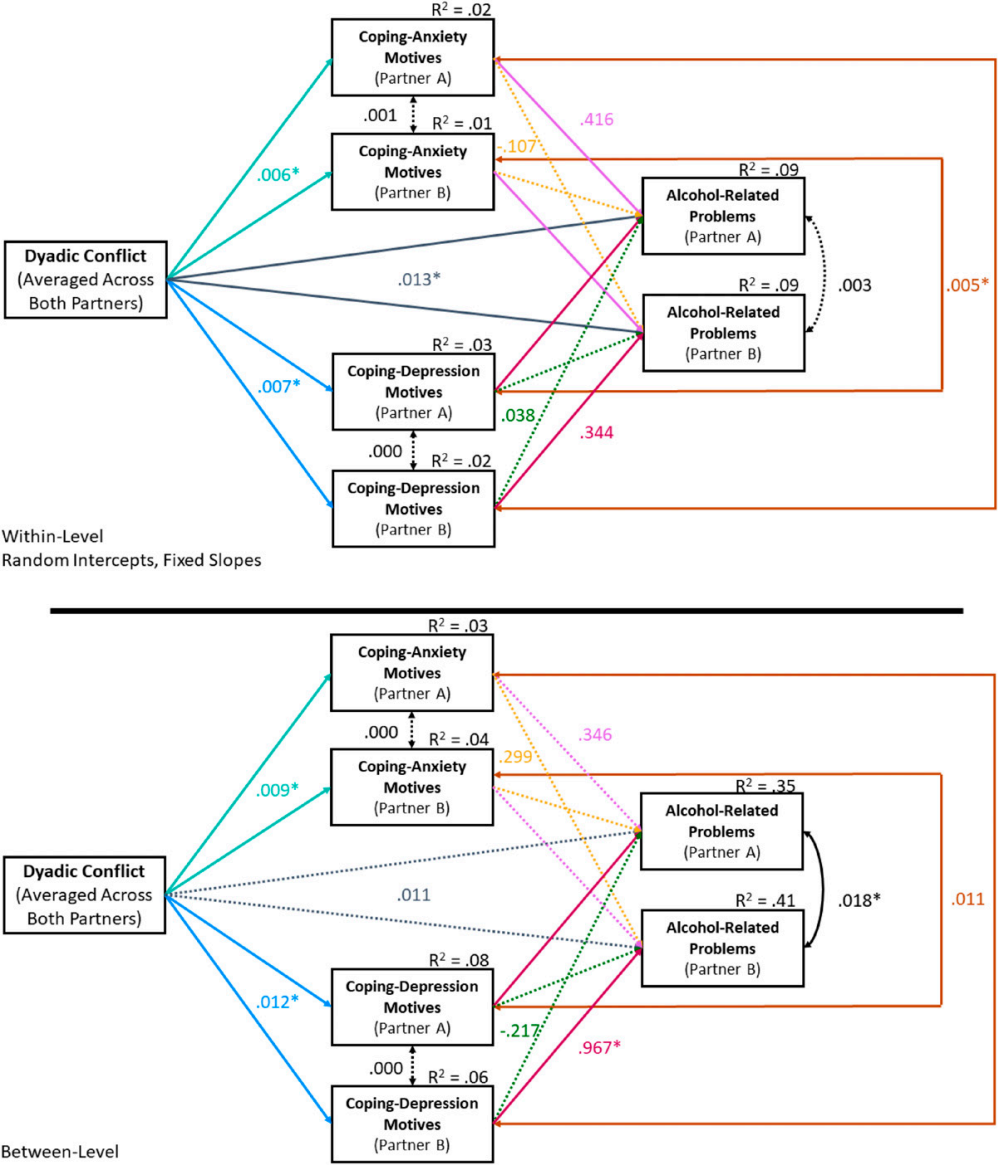
Results of the multilevel structural equation model predicting alcohol-related problems. *Note.* Solid lines indicate significant paths, dashed lines indicate nonsignificant paths. Rectangles indicate manifest variables. Single-headed arrows indicate paths. Double-headed arrows indicate covariances. Coefficients are unstandardized and paths were constrained to equality across both partners. R^2^ values are indicated in the upper right-hand corner of endogenous variables. See https://osf.io/krs3v/for full output.

Excellent model fit was defined as follows: root-mean-square error of approximation (RMSEA) < .06, standardized root-mean-square residual (SRMR) < .08, and comparative fit index (CFI) and Tucker–Lewis index (TLI) > .95 (Hu & Bentler, 1999; Kline, 2015). Finally, internal consistency was examined at the between- and within-subject levels using a multilevel adaptation of Cronbach’s alpha (Geldhof et al., 2014). The hypothesized model fit well, χ^2^(20) = 32.51, *p <.05*; RMSEA = .03; SRMR (within) = .07; SRMR (between) = .06, CFI = .98, TLI = .95. The unstandardized path coefficients and the associated *R*^*2*^ values are presented in Figure 3, with indirect effects presented in Table 3.

**Table 3.** Tests of Indirect Effects for the Multiple Mediation Model with CDM and CAM.

Predictor (X)	Mediator (M)	Outcome (Y)	CI (within-subjects)	CI (between-subjects)
Conflict	Actor’s CDM	Actor’s RAPI	^a^ [.000, .004]*	^a^ [.002, .021]*
Conflict	Actor’s CDM	Partner’s RAPI	[−.001, .002]	[−.009, .003]
Actor’s CDM	Actor’s RAPI	Partner’s RAPI	[−.002, .004]	[.004, .031]*
Actor’s CDM	Partner’s RAPI	Actor’s RAPI	[−.001, .001]	[−.013, .005]
Conflict	Actor’s CAM	Actor’s RAPI	[.000, .005]*	[−.001, .007]
Conflict	Actor’s CAM	Partner’s RAPI	[−.002, .001]	[−.002, .007]
Actor’s CAM	Actor’s RAPI	Partner’s RAPI	[−.002, .005]	[−.002, .015]
Actor’s CAM	Partner’s RAPI	Actor’s RAPI	[−.002, .001]	[−.003, .014]

*Note.* Indirect effects were derived using unstandardized coefficients. CI = Confidence Interval (95% level of confidence); RAPI = Rutgers Alcohol Problem Index; CDM = coping with depression motives; CAM = coping with anxiety motives.

^a^Confirmatory analyses; Bold CIs with * identify significant indirect effects whose 95% CIs do not cross zero.

#### Confirmatory analyses

At both the between- and within-subject levels, conflict significantly predicted CDM and CAM. At the within-subjects level, significant actor effects were found where CDM significantly predicted alcohol-related problems in the same individual; partner effects were not significant. Tests of indirect effects revealed that CDM significantly mediated the link between conflict and alcohol-related problems.

#### Exploratory findings

At the within-subjects level, conflict significantly predicted alcohol-related problems after controlling for all other variables; no effects were found at the between-subjects level. At the within-subjects level, significant actor effects were found where CAM significantly predicted alcohol-related problems in the same individual; partner effects were not significant. Tests of indirect effects revealed that CAM significantly mediated the link between conflict and alcohol-related problems. No other significant indirect effects were found at the within-subjects level. At the between-subjects level, significant actor effects were found where CDM (and not CAM) significantly predicted alcohol-related problems in the same individual; partner effects were not significant. At the between-subjects level, tests of indirect effects revealed that CDM (but not CAM) significantly mediated the link between conflict and alcohol-related problems. Indirect effects tests also revealed that the actor’s alcohol-related problems significantly mediated the link between the actor’s CDM and the partner’s alcohol-related problems (an indirect partner effect). No other significant indirect effects were found at the between-subjects level.

At the within-subjects level, the correlated error terms between the actor’s and partner’s alcohol-related problems were not significant (*B* = 0.003, *p* = .47). Although the correlated error terms between the actor’s and partner’s CDM (*B* < 0.001, *p* = .88) and CAM (*B* = 0.001, *p* = .30) were also not significant, CDM and CAM were strongly related within the same individual (*B* = 0.005, *p* < .001). The correlated error terms between alcohol-related problems were significant at the between-subjects level (*B* = 0.018, *p* = .001). Although the correlated error terms between partner’s CDM (*B* < 0.001, *p* = .55) and CAM (*B* < .0001, *p* = .96) remained unrelated, CDM and CAM maintained their relationship within the same individual (*B* = 0.011, *p* < .001). In general, effect sizes at the within-subjects level were small with about 1–3% of the variance explained in both coping motives, and 9% of the variance explained in alcohol-related problems. At the between-subjects level, for both coping motives, effect sizes were small with 3–8% of variance explained in both coping motives. For alcohol-related problems, however, a large amount of the variance was explained by conflict and coping motives combined (35–41%).

### Supplementary Analyses

Upon reviewer request, we included a few additional unplanned analyses. We tested the same model depicted in Figure 2, replacing alcohol problems with total volume of consumption using the STLFB measure. As in prior analyses, alcohol consumption was log_10_ transformed prior to analysis. The hypothesized model fit well, χ^2^(20) = 37.24, *p = .01*; RMSEA = .04; SRMR (within) = .06; SRMR (between) = .06, CFI = .97, TLI = .94. Broadly speaking, variables were weaker predictors of alcohol consumption (R^2^ = .12–.15) than of alcohol problems (R^2^ = .35–.41). In terms of hypothesis tests, the following differences from the analyses with alcohol problems as the outcome were noted: (a) Actor effects from CDM to alcohol consumption were non-significant at both the between- and within-subjects levels; (b) Actor effects from CAM to alcohol consumption became statistically significant and positive at the between-subjects level; and (c) The correlated error term for alcohol consumption in each partner became statistically significant and positive. All other paths showed the same pattern of statistical significance and direction of effects as the model with the alcohol problems measure as the outcome. All unstandardized path coefficients and the associated *R*^*2*^ values are presented in Supplementary Figure 1, with indirect effects presented in Supplementary Table 1.^5^

We also ran a supplementary analysis where we entered sex as a between-subjects covariate (i.e., a predictor of all variables in Figure 3). Note that sex cannot predict within-subjects variables, due to how the variance was partitioned for the multilevel model (i.e., it is a between-subjects variable that does not vary over time). In this model, sex was not significantly related to any other variable (all *p*s > .05). Moreover, all the coefficients in Figure 3 retained the same pattern of statistical significance and direction of effects when sex was included as a covariate. The raw output of this supplementary analysis can be found on our OSF page: https://osf.io/krs3v/

### Discussion

In both Lambe et al. (2015) and the present paper, relationship conflict positively predicted both CAM and CDM. That is, conflict in close relationships predicts drinking to cope at the between-person and within-person levels. Thus, the findings for the link of conflict to both types of coping drinking motives were successfully replicated. Where the results diverge is at the coping motives to alcohol problems stage. In Lambe et al. (2015), there was an actor effect only for CDM predicting alcohol problems, whereas the present paper finds actor effects for both CDM and CAM, at the within-subjects level. At the between-subjects level, there was substantial collinearity—that is, CAM and CDM were more strongly correlated with each other. This is not surprising, as depression and anxiety have been well-known to be comorbid for decades (Stavrakaki & Vargo, 1986). Thus, in the original Lambe et al. (2015) paper, CDM and CAM both predicted alcohol problems, but when entered together, the results became inconclusive. The present paper had a larger sample size, and was better able to handle the collinearity issue, and thus we observed CDM emerging as statistically significant predictor at the between-subjects level, even when controlling for CAM. Thus, the actor effects from CDM to alcohol problems were successfully replicated from Lambe et al. (2015). Additionally, supplementary analyses suggest that these findings persist even when controlling sex, and that coping motives and conflict are stronger predictors of alcohol-related problems than of volume of alcohol consumption. However, in the present paper relative to Lambe et al., we saw a stronger relationship between CAM and alcohol problems at the within-subjects level, which bears further consideration.

When conflict is present, friends may be turning to maladaptive coping mechanisms (i.e., coping drinking) for dealing with feelings of anxiety over time. There is some evidence to suggest that anxiety and depression are each associated with distinct patterns of alcohol use (Grant, Stewart, O'Connor, et al., 2007). Given that the mediation occurred in the current study with friendship pairs, and not when romantic couples were assessed, the type of relationship may explain the difference in CAM as a mediator between conflict and alcohol-related problems. Sex differences are reported in response to conflict, where men respond to conflict with both depression and anxiety, whereas women respond with only anxiety (El-Sheikh et al., 2013). Considering that the romantic couples study involved similar numbers of men and women, and this study was comprised of more women (66.1%), perhaps these differences are attributable to the greater proportion of women in the present study. Given that depression and anxiety may be associated with different alcohol-use patterns (Grant, Stewart, O’Connor, et al., 2007), the mechanisms and contextual factors underlying depression-related drinking may be distinct from those underlying anxiety-related drinking (Grant & Stewart, 2007; Grant, Stewart, & Birch, 2007; Grant et al., 2009). Of interest, supplementary analyses suggested that CAM (but not CDM) were positively associated with volume of drinking when both motives were entered as simultaneous mediators. This makes some sense, given the well-known anxiolytic properties of alcohol (Stewart et al., 2016).

Further, the two studies used different measurement timeframes; whereas Lambe et al. (2015) followed romantic couples over four 1-week study waves, the current study followed friendship pairs over four 1-month study waves. The longer intervals between waves in the current study may have provided greater opportunity for CAM to exert effects on alcohol-related problems over time (i.e., at the within-subjects level). Consistent with our finding of CAM as a predictor of alcohol-related problems at the within-subjects level (over time), Grant, Stewart, O'Connor, et al. (2007) identified that only CAM were predictive of alcohol-related problems prospectively over a mean follow-up interval of 94.8 days, when usual alcohol use was controlled. Thus, CAM may be more relevant for alcohol problems over longer time frames.

Similar to the findings of Lambe et al. (2015), no direct partner effects were found, suggesting that coping motives in response to interpersonal conflict may influence alcohol problems at only the individual level. An indirect partner effect observed by Lambe et al. (2015) was replicated, with the relationship between an individual’s CDM and their partner’s alcohol-related problems being mediated by the individual’s own alcohol-related problems at the between-subjects level. Therefore, although no direct influence of a friend’s CDM on the individual’s alcohol-related problems was found, the presence of these motives in the friend indirectly led to alcohol-related problems for the individual by way of first influencing the friend’s alcohol-related problems which in turn influenced the individual’s alcohol-related problems (potentially via modeling of maladaptive drinking; see Muyingo et al., 2020).

At both the within- and between-subject levels, conflict was a significant predictor of CDM and CAM, and an indirect predictor of alcohol-related problems. At the within-subjects level, both CDM and CAM subsequently predicted alcohol-related problems. These findings are broadly consistent with previous research with romantic couples (Lambe et al., 2015), and support predictions of drinking motives theory (Cooper et al., 2016). These findings suggest that emerging adults may use alcohol to cope with feelings of depression and anxiety following conflict with their friends, a maladaptive behavior that leads to alcohol-related problems. Further, over time, alcohol-related problems may occur following conflict with friends. Coping with both feelings of depression and anxiety appear to be important mechanisms through which friendship conflict leads to alcohol-related problems over time, whereas only CDM appears to be an important mechanism through which conflict leads to alcohol-related problems at the between-subjects level.

In adolescents, it has been found that peer drinking motives influence individual drinking motives (Kuntsche & Stewart, 2009). It was therefore unexpected in the present study that coping motives were unrelated between friends over time. However, this finding replicated the work of Lambe et al. (2015) that also found no partner effects for coping motives within romantic couples. The lack of partner effects found in both studies may be related to contextual factors for coping drinking, in that individuals drinking to cope with negative affect are more likely to do so alone at home than with a friend or partner (Cooper, 1994). Therefore, drinking to cope with feelings of anxiety or depression resulting from interpersonal conflict may be a relatively solitary process that does not influence a friend’s coping motives. This is supported by our supplementary analyses, which demonstrate a positive correlated error term for volume of consumption, but not for alcohol problems—that is, partners influence each other’s drinking habits, but may not be similar in their levels of alcohol-related problems.

### Clinical Implications

These results extend the Lambe et al. (2015) findings with emerging adults’ romantic partners to the role of coping drinking motives mediating the relationship between friendship conflict and both alcohol-related problems and alcohol volume in emerging adults. Interventions for emerging adults’ heavy and problem drinking should consider the influence of conflict within close friendships and its impact on tendencies to drink to cope with both depression and anxiety. Further, interventions may include strategies for reducing CDM and CAM to reduce associated heavy drinking and alcohol-related problems. Considering that our results showed that alcohol-related problems and alcohol volume changed systematically in the same direction between friends, dyadic-level interventions may be useful to address both friends’ alcohol-related problems and associated influences, like conflict.

### Limitations and Future Directions

This study is not without its limitations. The sample was primarily White university students who were less than 21 years old, which limits generalizability. Additionally, there may have been some self-selection of dyads in the participation pool (i.e., people who agree to participate in research studies may be different from those who do not). Despite rejecting the null hypothesis, the magnitudes of relationships were often small. Through supplementary analyses, Lambe et al. (2015) identified sex differences where the mediation of CDM between conflict and alcohol-related problems was significant only for women. Due to the relatively small sample size of men in the current study, sex differences were not explored, although our results did hold when sex was controlled in supplementary analyses. Future research with a larger sample size and more male participants might examine sex differences in friendship dyads to determine if these sex differences extend to other forms of interpersonal conflict. Statistical power for the within-subjects path from dyadic conflict to CAM was low; thus, non-significant results here may reflect a Type II error. Data were collected retrospectively at the end of every 30-day period. Future studies may examine these variables daily via ecological momentary assessment for more accurate in-time measurement, as drinking to cope may occur relatively quickly following conflict. This study focused on the relationship between conflict and alcohol-related problems. It may be interesting to explore the role of conflict resolution, and whether this has any influence on associated drinking motives (Rodriguez et al., 2019). The study sample consisted of emerging adult undergraduate friendship pairs, with 66.1% of the sample being women. The findings may not be generalizable to other samples (e.g., adolescents, older friends, and opposite-sex friendships). At least one member of the dyad was always a first-year undergraduate student; these students may be particularly vulnerable to friendship conflict due to the social difficulties of transitioning to university so these results may not generalize to all types of friendships. Nonetheless, it is interesting that these findings occur in comparatively new friendships, which may be a more socially tumultuous time as friends test new boundaries and limits in their friendship. Finally, although existing theory suggests that other drinking motives should be unrelated to conflict, future research may explore these other motives to establish the specificity of the present findings to the coping motives.

Our analytic strategy has two notable limitations. First, our use of a fixed slopes, random intercepts model assumes compound symmetry for the longitudinal correlated residuals, which may be overly simplistic. Though more complex, other analysts are developing approaches to incorporate AR(1) correlated residual structures, which might fit the data better (Gistelinck & Loeys, 2019). Second, our use of log transformations trades interpretability for robustness. That is, by transforming the raw data, our residuals look closer to a normal distribution and thus the estimates for *p*-values will likely be more trustworthy. However, because coefficients are difficult to interpret in the log scale, it is more difficult to assess the magnitude of effects.

## Conclusions

This study replicated and extended the work of Lambe et al. (2015), confirming that in undergraduate friendships, the relationship between friendship conflict and alcohol-related problems is mediated by CDM at the within-subjects level. Further, the replication extended to CDM mediating friendship conflict and alcohol-related problems at the between-subject level. Exploratory analyses also revealed indirect partner effects where CDM in the friend lead to alcohol-related problems in the individual by way of first influencing the friend’s alcohol-related problems. Unique to our results was the finding that in friendships, the relationship between conflict and alcohol-related problems is also mediated by CAM at the within-subjects level. The findings from this work are important in contributing understanding of conflict and coping drinking motives to intervention efforts for preventing and intervening with alcohol-related problems in emerging adults.

## Supplemental Material

sj-pdf-1-eax-10.1177_21676968211060945 – Supplemental Material Friendship Conflict, Drinking to Cope, and Alcohol-Related Problems: A Longitudinal Actor-Partner Interdependence Model Click here for additional data file. Supplemental Material, sj-pdf-1-eax-10.1177_21676968211060945 for Friendship Conflict, Drinking to Cope, and Alcohol-Related Problems: A Longitudinal Actor-Partner Interdependence Model by Sean P. Mackinnon, Michelle E. Tougas, Ivy-Lee L. Kehayes and Sherry H. Stewart in Emerging Adulthood

## Notes

**Open Practices:** The analysis code and materials used in this manuscript are openly available (https://osf.io/krs3v/) The raw data contained in this manuscript are not openly available due to privacy restrictions set forth by the institutional research ethics board but can be obtained from the corresponding author following the completion of a privacy and fair use agreement. No aspects of the study were pre-registered.

**Notes:** 1. Data collection in this study continued until the timeline for our funding ran out. However, we conducted a power simulation prior to our analyses. Broadly, this analysis suggested that the sample size was adequate to detect similarly-sized effect sizes to Lambe et al. (2015). See the online supplementary materials for more details.

2. The full questionnaires for the materials listed here can be found at https://osf.io/krs3v/. Other variables measured in the study are available upon request.

3. When the log transformations are not used, the pattern of statistical significance and direction of effects noted in Figure 3 do not change. However, the overall model fit indices are considerably poorer when the log transformations are not used: χ^2^(20) = 52.85, *p < .001*; RMSEA = .05; SRMR (within) = .11; SRMR (between) = .09, CFI = .94, TLI = .88. Simulation work by Gao et al. (2020) suggests that, in the presence of non-normality, robust fit indices tend to erroneously reject models as fitting poorly. Thus, we believe the log transform is useful in the present case. The raw output of this analysis available at https://osf.io/krs3v/.

4. In the current study, the RAPI assessed alcohol-related problems over the past 30 days. In past studies (e.g., Lambe et al., 2015), the RAPI has been used to assess a 1-week time frame. Due to these differences in time frame, the means are not directly comparable, and it was expected that the 30-day RAPI scores would be higher.

5. Readers should note that this analysis overlaps somewhat with another paper that analyzes the same dataset (Kehayes et al., 2021). Specifically, they tested the bivariate relationships between drinking motives and alcohol quantity and frequency separately. Though these analyses differ slightly (i.e., using volume instead of separate quantity/frequency indices; adding conflict and both motives as simultaneous mediators), readers interested in this topic are encouraged to also refer to Kehayes et al. (2021) for further analyses on alcohol consumption using this dataset.
