# Supplementary material for: Friendship Conflict, Drinking to Cope, and Alcohol-Related Problems: A Longitudinal Actor-Partner Interdependence Model
Source: Emerg Adulthood. 2022 Mar 11;10(3):595–608. doi: 10.1177/21676968211060945 (PMC9082978; doi:10.1177/21676968211060945)
Supplement: sj-pdf-1-eax-10.1177_21676968211060945 – Supplemental Material Friendship Conflict, Drinking to Cope, and Alcohol-Related Problems: A Longitudinal Actor-Partner Interdependence Model [file sj-pdf-1-eax-10.1177_21676968211060945.pdf]

Power Analysis

Power analysis used a Monte Carlo simulation in Mplus 8.0. We first ran the proposed model on the replication target's data (Lambe et al., 2015), saved the model's parameter estimates and variances, and input these values as the population parameters in the power analysis. We ran a simulation assuming *N* dyads = 152 (i.e., final cluster size after assuming 80% of possible observations being usable) and item-level missingness of 1%. Thus, this is a power analysis using population parameters from the replication target, and the sample size from the present study. Statistical power for confirmatory paths is as follows: dyadic conflict predicting CDM (within = 58%; between = 99%), dyadic conflict predicting CAM (within 78%; between 95%), CDM predicting RAPI (within = 97%). Thus, statistical power is adequate for all confirmatory paths except for the within-subjects relationship between dyadic conflict and CDM. Thus, it must be acknowledged that Type II error rates are high for this latter relationship. Full results from the power analysis can be found at https://osf.io/krs3v/.

Supplementary Figure 1

Results of the Multilevel Structural Equation Model Predicting Alcohol Consumption

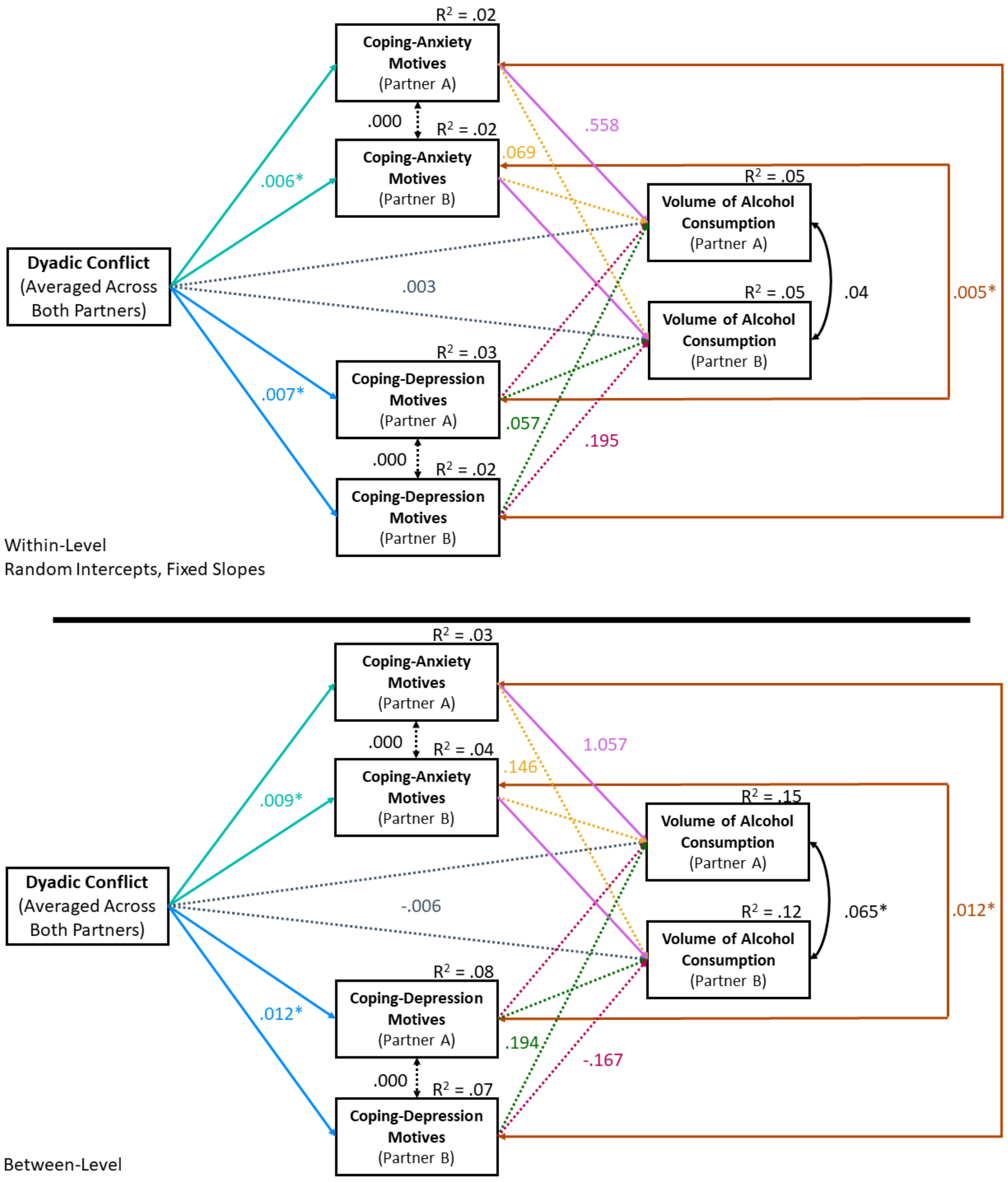

Note. Bold lines indicate significant paths, dashed lines indicate nonsignificant paths. Rectangles indicate manifest variables. Single-headed arrows indicate paths. Double-headed arrows indicate covariances. Coefficients are unstandardized and paths were constrained to equality across both partners. R^2 values are indicated in the upper right-hand corner of endogenous variables. See https://osf.io/krs3v/ for full output.

Supplementary Table 1

Tests of Indirect Effects for the Multiple Mediation Model For Alcohol Consumption

Predictor (X)	Mediator (M)	Outcome (Y)	CI (within-subjects)	CI (between-subjects)
Conflict	Actor's CDM	Actor's STLFB	[-.001, .004]	[-.010, .006]
Conflict	Actor's CDM	Partner's STLFB	[-.002, .003]	[-.006, .010]
Actor's CDM	Actor's STLFB	Partner's STLFB	[-.009, .024]	[-.054, .032]
Actor's CDM	Partner's STLFB	Actor's STLFB	[-.001, .015]	[-.032, .057]
Conflict	Actor's CAM	Actor's STLFB	**[.000, .006]***	**[.000, .019]***
Conflict	Actor's CAM	Partner's STLFB	[-.001, .002]	[-.005, .007]
Actor's CAM	Actor's STLFB	Partner's STLFB	**[.009, .035]***	**[.026, .112]***
Actor's CAM	Partner's STLFB	Actor's STLFB	[-.007, .013]	[-.031, .050]

Note. Indirect effects were derived using unstandardized coefficients. CI = Confidence Interval (95% level of confidence); STLFB = Self-reported Timeline Follow-Back; [1]Confirmatory analyses; Bold CIs with * identify significant indirect effects whose 95% CIs do not cross zero.
